# Plant signaling networks involving Ca^2+^ and Rboh/Nox-mediated ROS production under salinity stress

**DOI:** 10.3389/fpls.2015.00427

**Published:** 2015-06-10

**Authors:** Takamitsu Kurusu, Kazuyuki Kuchitsu, Yuichi Tada

**Affiliations:** ^1^School of Bioscience and Biotechnology, Tokyo University of TechnologyHachioji, Japan; ^2^Department of Applied Biological Science, Tokyo University of ScienceNoda, Japan; ^3^Research Institute for Science and Technology, Tokyo University of ScienceNoda, Japan

**Keywords:** Ca^2+^-permeable channels, NADPH oxidases (Noxs), osmotic stress, reactive oxygen species (ROS), salinity stress

## Abstract

Salinity stress, which induces both ionic and osmotic damage, impairs plant growth and causes severe reductions in crop yield. Plants are equipped with defense responses against salinity stress such as regulation of ion transport including Na^+^ and K^+^, accumulation of compatible solutes and stress-related gene expression. The initial Ca^2+^ influx mediated by plasma membrane ion channels has been suggested to be crucial for the adaptive signaling. NADPH oxidase (Nox)-mediated production of reactive oxygen species (ROS) has also been suggested to play crucial roles in regulating adaptation to salinity stress in several plant species including halophytes. Respiratory burst oxidase homolog (Rboh) proteins show the ROS-producing Nox activity, which are synergistically activated by the binding of Ca^2+^ to EF-hand motifs as well as Ca^2+^-dependent phosphorylation. We herein review molecular identity, structural features and roles of the Ca^2+^-permeable channels involved in early salinity and osmotic signaling, and comparatively discuss the interrelationships among spatiotemporal dynamic changes in cytosolic concentrations of free Ca^2+^, Rboh-mediated ROS production, and downstream signaling events during salinity adaptation *in planta*.

## Introduction

Soil salinity impairs plant growth and development and is an important factor limiting crop productivity and yield worldwide (Rengasamy, 2006). Salinity stress reduces water potential, thereby preventing water uptake by roots and provoking a set of responses similar to those of a water deficit. Thus, by causing osmotic stress, salinity provokes the stomatal limitation of photosynthesis, loss of turgor, enhanced photorespiration, and excess production of ROS (Chaves et al., 2009). The ionic component of salinity stress is attributed to the direct toxic effects of Na^+^ and imbalances in the homeostasis of other ions such as K^+^ and Ca^2+^ (Munns and Tester, 2008).

To mount an effective response to cope with salinity stress, land plants have developed the ability to sense both osmotic stress and Na^+^ (Deinlein et al., 2014). Plants exhibit a rapid increase in the cytosolic Ca^2+^ concentration within seconds of being exposed to NaCl or mannitol (Knight et al., 1997). The Ca^2+^ rise originated within the roots (Tracy et al., 2008) is propagated systemically (Choi et al., 2014), and occurs in several cell types (Kiegle et al., 2000).

Reactive oxygen species such as $O_{2}^{\cdot -}$ (superoxide anion radical), H_2_O_2_ (hydrogen peroxide), and OH^⋅^ (hydroxyl radical) are highly toxic substances produced through aerobic respiration and photosynthesis that oxidize various biomolecules such as DNA, proteins and lipids, and disrupt the cell redox state. Therefore, plants have developed various systems to scavenge ROS (Choudhury et al., 2013). Direct exposure of plant tissues to H_2_O_2_ activates antioxidant enzymes as well as the expression of the corresponding genes (Mylona et al., 2007). ROS are also enzymatically produced and act as signaling molecules in plant responses to abiotic and/or biotic stresses (Kärkönen and Kuchitsu, 2015).

Respiratory burst oxidase homologs has been identified as ROS-producing Noxs that generate $O_{2}^{\cdot -}$ by oxidizing NADPH and transferring an electron to oxygen (Sumimoto, 2008). Noxs are rapidly activated in response to various stimuli to induce ROS production and are involved in the regulation of a wide range of physiological functions in plants (Marino et al., 2012). Rbohs play central roles in the Ca^2+^-ROS signaling network triggered by their phosphorylation during stress adaptation (Takeda et al., 2008; Kimura et al., 2012; Gilroy et al., 2014).

We here review molecular mechanisms and roles of PM Ca^2+^-permeable channels as putative osmosensors and enzymatic ROS production involved in early salinity and osmotic responses, and discuss the interrelationships among dynamic changes in [Ca^2+^]_cyt_, ROS production, downstream signaling events during salinity sensing and adaptation in plants including halophytes.

## Candidates for Plant Ca^2+^-Permeable MS Channels as Osmosensors

Mechanical stimuli, such as touch, bending, and barriers for growth as well as osmotic potential changes, all elicit rapid and transient increases in [Ca^2+^]_cyt_. Expression of cellular Ca^2+^-binding regulatory proteins including CaM and CML are induced by mechanical stimuli (Braam and Davis, 1990). Early responses to salinity stress reflect turgor changes and involve Ca^2+^-mediated signaling (Donaldson et al., 2004). PM Ca^2+^-permeable MS channels are suggested to sense salinity stress-triggered osmotic potential change. The following protein families are suggested as putative plant PM Ca^2+^-permeable MS channels as osmosensors.

### OSCA1

OSCA1 was recently identified as a novel putative PM hyper-osmolality-gated Ca^2+^-permeable channel in guard cells and roots of *Arabidopsis* (Yuan et al., 2014). OSCA1 comprises nine transmembrane α-helices, but shows no significant similarity to known ion channels. Electrophysiological analyses of OSCA1 expressed in HEK293 cells showed its hyper-osmolality-gated Ca^2+^-permeable channel activity (Yuan et al., 2014). In *osca1*, early osmotic signaling events in guard cells were impaired and downstream adaptation responses against osmotic stress were attenuated (Yuan et al., 2014).

### MCA Family

The yeast Mid1 protein shows MS channel activity when expressed in mammalian cells (Kanzaki et al., 1999). MCAs were identified in *Arabidopsis* based on their ability to complement the yeast mutant *mid1* (Nakagawa et al., 2007). MCAs comprise at least one transmembrane domain and contain an EF hand, but show no significant similarity to known ion channels. All MCAs are localized to the PM and mediate Ca^2+^ uptake, with the N-terminal and EF hand regions being necessary structures for transport. MCAs are present in all land plants, including ferns and mosses, but not in algae, animals, protists, or fungi, suggesting that the function of MCAs is fundamental to land plants (Kurusu et al., 2013).

Ca^2+^ current induced by trinitrophenol, a potent compound to generate membrane distortion to activate MS channels, is enhanced in the *MCA1*-overexpressing cells (Nakagawa et al., 2007), and *MCA1* and *MCA2* expressed in *Xenopus* oocytes showed MS cation channel activity (Furuichi et al., 2012). Touch response in the primary root is impaired in *mca1*, while *mca2* showed lower root Ca^2+^ uptake (Nakagawa et al., 2007; Yamanaka et al., 2010), suggesting that MCA1 and MCA2 play different physiological roles. Hypo-osmotic shock-induced PM Ca^2+^ influx and ROS production are partially impaired in *OsMCA1*-suppressed rice cultured cells (Kurusu et al., 2012a). Tobacco MCA homologs are localized at the Hechtian strand that connects PM and cell wall (Kurusu et al., 2012b). These findings suggest that MCAs are involved in hypo-osmotic stress-induced PM Ca^2+^ influx (Kurusu et al., 2013). MCA1 and MCA2 form a tetramer (Nakano et al., 2011; Shigematsu et al., 2014), suggesting that MCA proteins may function as a tetramer to form a Ca^2+^-permeable pore.

### MSL Family and Others

Genome-wide screening to search for plant MS channels homologous to eukaryotic and prokaryotic MS channels identified the MSL protein family (Haswell et al., 2011). MSLs are present in land plants, and electrophysiological studies suggest that MSL9 and MSL10 permeate Cl^-^ rather than Ca^2+^ (Haswell et al., 2008). It remains unknown whether any of MSLs are involved in the mechanical stimuli-induced Ca^2+^ influx as Ca^2+^-permeable channels.

Piezo proteins in mouse and *Drosophila* are also pore-forming subunits of MS channels and respond to mechanical stimuli. Many eukaryotic species including plants but not yeast and bacteria have a single Piezo protein (Coste et al., 2010). Functional roles of plant Piezo protein have not yet been reported.

An *Arabidopsis* histidine kinase HK1 may share a similar function with yeast SLN1 as an osmosensor (Urao et al., 1999; Wohlbach et al., 2008). It is of interest to determine whether any of these putative MS channels or osmosensors interacts with each other physically or functionally to monitor mechanical stimuli under salinity stress.

## Plant Signaling Networks Involving Ca^2+^ and ROS under Salinity Stress

### Physiological Roles of Rboh/Nox in Early Salinity Responses in *Arabidopsis*

Reactive oxygen species are highly toxic substances that oxidize various biomolecules. Generation of OH^⋅^ has significant implications to cell metabolism and causes lipid peroxidation (Apel and Hirt, 2004). K^+^ deficiency causes an increase in $O_{2}^{\cdot -}$ production by Rboh/Nox in root cells, and enhanced activation of antioxidant enzymes under salinity stress involves cytosolic K^+^ retention, suggesting that optimizing the K^+^ nutritional status may reduce the toxic effect of ROS by regulating Nox activity, and is essential for plant performance against salinity (Shabala and Pottosin, 2014).

Exposure to salinity activates the SOS pathway, leading to Ca^2+^-dependent activation of SOS1, a PM Na^+^/H^+^ exchanger that enables adaptation through Na^+^ eﬄux (Shi et al., 2000; Chung et al., 2008). Expression of *SOS1* is induced under salinity stress, which requires activation of the Ca^2+^ sensor SOS3/CBL4 (calcineurin B-like 4) (Shi et al., 2000). Nox-mediated ROS production stabilizes *SOS1* transcripts (Chung et al., 2008). Growth of better-adapted secondary roots is impaired in *sos1* (Huh et al., 2002) and involves $O_{2}^{\cdot -}$ production, possibly by Rboh/Nox (Roach and Kranner, 2011).

*Arabidopsis* contains 10 Nox genes, *RbohA-J*, which exhibit different patterns of expression and in response to environmental factors (Marino et al., 2012). Expression of *RbohD* and *RbohF* are highly induced under salinity stress (Ma et al., 2012). An *RbohF* mutant showed strong Na^+^ hypersensitivity in its shoots and lacked ROS accumulation in its vasculature (Jiang et al., 2012), suggesting that RbohF plays a role in the regulation of xylem loading of Na^+^ to protect leaves from salinity stress in *Arabidopsis*. ROS production by Rboh/Nox is also suggested to be coordinated by signaling pathways involving phospholipid and actin in salinity stress responses, and is required for heme oxygenase-mediated salt acclimation signaling (Xie et al., 2011).

### The Interrelationships between Ca^2+^ Influx and Rboh/Nox-Mediated ROS Production in Early Salinity Responses

Plant Rbohs have two N-terminal EF-hand motifs. A heterologous expression system based on HEK293T cells is effective to quantitatively evaluate (Ogasawara et al., 2008) and reconstitute the ROS-producing activity of Rbohs (Drerup et al., 2013; Kimura et al., 2013). Rbohs are basically synergistically activated by the Ca^2+^-binding to the EF-hand motifs and phosphorylation (Ogasawara et al., 2008). Protein phosphorylation is a prerequisite for the Ca^2+^-dependent activation of Rbohs (Kimura et al., 2012). *Arabidopsis* CIPK26, binds to RbohF *in planta* (Kimura et al., 2013) and in the presence of PM-localized Ca^2+^ sensor proteins CBL1/CBL9, CIPK26 enhances the activity of RbohF (Drerup et al., 2013). Rbohs are phosphorylated and activated by two families of Ca^2+^-dependent protein kinases (CBL-CIPK complexes and CDPKs; Kobayashi et al., 2007; Drerup et al., 2013; Dubiella et al., 2013), and two families of Ca^2+^-independent protein kinases (RLCKs and SnRK2s; Sirichandra et al., 2009; Kadota et al., 2014).

Reactive oxygen species-activated Ca^2+^-permeable channels and Rbohs have been suggested to constitute a positive feedback mechanism that enhances Ca^2+^ and ROS signals in root cells (Takeda et al., 2008). RbohC/RHD2 affects mechanical stress-induced ROS generation, and regulates *Arabidopsis* root hair elongation in a Ca^2+^-dependent manner (Takeda et al., 2008; Monshausen et al., 2009). Hyperpolarization- and depolarization-activated PM Ca^2+^-permeable channels may play roles in salinity-induced [Ca^2+^]_cyt_ increase (Tracy et al., 2008).

Salinity-induced elevation of [Ca^2+^]_cyt_ corresponds to PM Ca^2+^ influx as well as Ca^2+^ release from intracellular Ca^2+^ stores, and plays important roles in ROS signaling and salt tolerance (Shabala and Newman, 2000; Kader and Lindberg, 2010). Salinity-induced Ca^2+^ influx currents were markedly suppressed in the *rbohD/F* double mutant in *Arabidopsis*, implicating the roles of RbohD and RbohF in salt-stimulated [Ca^2+^]_cyt_ rise (Ma et al., 2012).

Plant annexins have been reported to form Ca^2+^-permeable channels in planar lipid bilayers (Laohavisit and Davies, 2011). *Arabidopsis* AtANN1 is localized at the PM and responsible for root epidermal PM Ca^2+^-permeable conductance, which is activated by extracellular OH^⋅^ (Lee et al., 2004; Laohavisit et al., 2012). High concentration of NaCl promotes extracellular formation of OH^⋅^ (Demidchik et al., 2010) and accumulation of AtANN1 in membranes (Lee et al., 2004), and promotes secondary root formation (Huh et al., 2002). AtANN1-mediated Ca^2+^ influx through the PM depends on extracellular Ca^2+^ and ROS, and the *atann1* mutant shows reduced secondary root formation and reduced activation of NaCl-induced transcription under salinity stress conditions (Laohavisit et al., 2013), suggesting a role of AtANN1 in root cell adaptation to salinity in *Arabidopsis*.

Salinity-induced Na^+^ accumulation in the cytosol triggers [Ca^2+^]_cyt_ elevation, leading to activation of Rboh/Nox (Lecourieux et al., 2006) and apoplastic H_2_O_2_ accumulation. By interacting with transition metals such as Fe in the cell wall, H_2_O_2_ forms OH^⋅^ (Kuchitsu et al., 1995; Rodrigo-Moreno et al., 2013). OH^⋅^ directly activates both outward-rectifying depolarization-activating K^+^ channels (GORKs) and K^+^-permeable NSCCs, resulting in K^+^ leakage from roots (Shabala and Pottosin, 2014). The absolute concentration of K^+^ is essential to confer salinity stress tolerance, and the loss of K^+^ is suggested to play a primary role in the activation of caspase-like proteases and PCD (Shabala, 2009).

Salinity also induces accumulation of polyamines. Polyamines could generate ROS as a substrate of amine oxidases in the apoplast (Kärkönen and Kuchitsu, 2015). Both OH^⋅^ and polyamines may provoke a substantial remodeling of PM conductance of cations and anions and affect Ca^2+^ signaling in plants including halophytes (Pottosin et al., 2014). Crosstalk between ROS and polyamines in the regulation of PM ion transport may reveal a novel function of ROS production as signaling molecules during salinity adaptation.

### A Ca^2+^-ROS Signaling Network in Osmotic Responses in Roots under Salinity Stress

Osmotic-shock induces Ca^2+^ influx followed by ROS production in many cell types in plants. The Ca^2+^ influx and Nox-mediated ROS production triggered by osmotic shock both require extracellular Ca^2+^, suggesting that PM Ca^2+^ influx is a prerequisite for ROS production (Beffagna et al., 2005).

*mid1*-complementing activities (MCAs), putative PM Ca^2+^-permeable MS channel components are suggested to play a role in the regulation of mechanical responses via signal transduction pathways dependent on Ca^2+^ and ROS (Kurusu et al., 2013). The overexpression of *OsMCA1* enhances Rboh/Nox-mediated ROS production in rice cultured cells (Kurusu et al., 2012a). MCA1 and ROS produced by RbohD and/or RbohF may play roles in the modulation of osmosensitive metabolic changes (Wormit et al., 2012). Overexpression of *MCAs* enhances the expression of touch-inducible gene *TCH3/CML12* encoding a CML in *Arabidopsis* (Nakagawa et al., 2007).

Apoplastic ROS production plays a key role in regulating cell wall metabolism, e.g., cross-linking of polysaccharides and glycoproteins to control cell wall rigidity (O’Brien et al., 2012; Kärkönen and Kuchitsu, 2015). H_2_O_2_ is also transported to the cytosol by PM aquaporins (Dynowski et al., 2008) to modify the cysteine residues of target proteins, thereby promoting redox signaling (Spoel and Loake, 2011).

**FIGURE 1:**
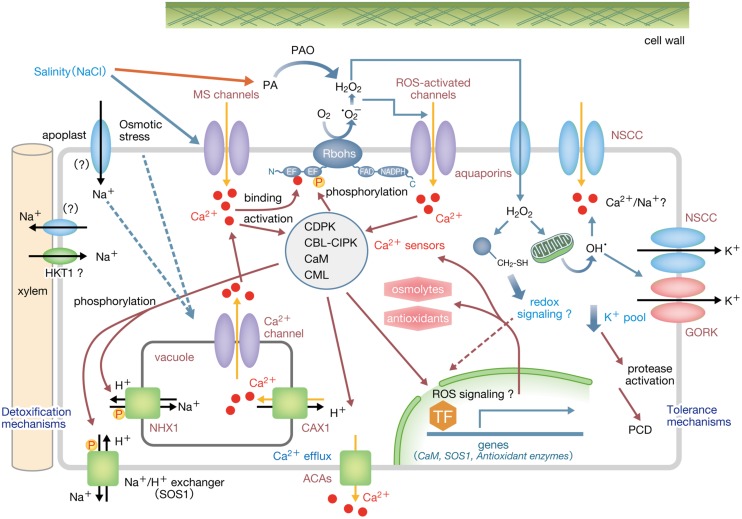
**A possible Ca^2+^-ROS signaling network involved in osmotic responses under salinity stress in root cells**. Salinity stress causes osmotic shock to trigger the activation of PM MS Ca^2+^-permeable channels and Ca^2+^ influx. Elevated [Ca^2+^]_cyt_ leads to activation of various Ca^2+^ sensor proteins including CaMs, CMLs, CBLs, CDPKs as well as the ROS-producing enzyme, Noxs/Rbohs that are synergistically activated by Ca^2+^ binding and phosphorylation by Ca^2+^-dependent protein kinases such as CDPKs and CBL–CIPK complexes. Elevated apoplastic ROS activates ROS-activated PM Ca^2+^-permeable channels to trigger further Ca^2+^ influx, while Ca^2+^-permeable endomembrane channels/transporters may also been activated to sustain further Ca^2+^ mobilization. Elevated [Ca^2+^]_cyt_ may trigger downstream events such as activation of SOS1 and NHX1, Na^+^/H^+^ exchangers that transport cytosolic Na^+^, regulation of xylem loading of Na^+^, induction of osmolytes and osmo-protective proteins, and the retention of cytosolic ion balance. Ca^2+^-dependent protein kinases activated by elevated [Ca^2+^]_cyt_ mediate downstream events including expression of genes encoding proteins such as SOS1, antioxidant enzymes, osmolytes-biosynthetic enzymes as well as CaMs as signal amplifiers. Elevated [Ca^2+^]_cyt_ is reverted by the orchestrated action of active transporters such as Ca^2+^ exchangers and Ca^2+^-ATPases at the PM and tonoplast. H_2_O_2_ is transported to the cytosol by PM aquaporins to modify the cysteine residues of target proteins, thereby promoting redox signaling to regulate gene expression. Broken arrows indicate hypothetical links. ACA, autoinhibited Ca^2+^-ATPase; PA, polyamine; PAO, polyamine oxidase.

These findings suggest the following initial PM signaling mechanism in response to osmo-stimulation under salinity stress (**Figure 1**): Salinity stress causes osmotic shock to trigger the activation of PM MS Ca^2+^-permeable channels and Ca^2+^ influx. Elevated cytosolic Ca^2+^ leads to the activation of various Ca^2+^ sensor proteins including CaMs, CMLs, CBLs, and CDPKs. Rbohs are synergistically activated by Ca^2+^-binding to the EF-hand motifs and phosphorylation to produce ROS. Elevated cytosolic Ca^2+^ and apoplastic ROS may also activate not only ROS-activated PM Ca^2+^-permeable channels including annexins but also as-yet-unidentified Ca^2+^-permeable channels/transporters localized at the endomembranes to sustain further Ca^2+^ mobilization. The signaling network dependent on Ca^2+^ and ROS may play a crucial role in regulating downstream events such as the Ca^2+^-dependent activation of Na^+^/H^+^ exchangers, SOS1 and NHX1 (Reguera et al., 2014), Na^+^ eﬄux from the cytosol, regulation of xylem loading of Na^+^ (de Boer and Wegner, 1997), Na^+^ exclusion from leaves (Sunarpi et al., 2005), induction of osmolytes and osmo-protective proteins, and the retention of cytosolic ion balance. Once this signaling is over, elevated [Ca^2+^]_cyt_ would be reverted by the orchestrated action of Ca^2+^ exchangers and Ca^2+^-ATPases at the PM and tonoplast (reviewed in Bose et al., 2011).

Calcium-dependent protein kinases (Boudsocq and Sheen, 2013) and CBL-CIPK complexes (Weinl and Kudla, 2009) are suggested to transduce the osmotic signal to regulate downstream posttranslational modification and gene transcription. Furthermore, transcription factors such as calmodulin-binding transcription activators (CAMTAs; Pandey et al., 2013) are suggested to be directly activated by Ca^2+^/CaM. Interestingly, a recent comparative expression study in glycophytes and halophytes revealed that the induction of Ca^2+^/CaM-like proteins such as CDPKs are clearly enhanced in halophytes against salinity stress (Xu et al., 2013). Such Ca^2+^-binding proteins may play roles as important ‘amplifier’ of initial PM Ca^2+^ influx and Rboh-mediated ROS production in salinity stress signaling.

Ca^2+^ signaling also plays a critical role in salinity stress-triggered systemic signaling. A cation channel TPC1 is reported to be involved in the propagation of salinity stress-triggered systemic Ca^2+^ wave in roots, and may also be contributing to whole-plant resistance to salinity stress in *Arabidopsis* (Choi et al., 2014). Ca^2+^-ROS waves involving TPC1 may elicit systemic molecular responses in target organs and contribute to whole-plant stress tolerance (Choi et al., 2014; Gilroy et al., 2014).

Hydrogen peroxide transported from the apoplast to the cytosol by PM aquaporins may also interact with $O_{2}^{\cdot -}$ generated in the mitochondria under salinity stress to generate OH^⋅^ in the cytosol by Fenton reaction, which could activate NSCCs from the cytosolic site (Rodrigo-Moreno et al., 2013). As NSCCs are permeable to Na^+^, Ca^2+^, and K^+^, this activation may enhance Na^+^ uptake and K^+^ loss, further causing the cytosolic K^+^/Na^+^ imbalance. The resultant K^+^ loss from the cell may induce activation of caspase-like proteases and leading to PCD. Moreover, OH^⋅^-induced activation of NSCCs may further boost Ca^2+^ uptake, providing an additional positive feedback loop.

## Possible Function of Initial ROS Production and Downstream Signaling Events during Stress Adaptation in Halophytes

The salt tolerance of halophytes is typically examined after long-term exposure to different NaCl concentrations (Tada et al., 2014). However, plant responses to salinity may be determined by rapid perception of salt shock that occurs within hours (Ellouzi et al., 2011; Wang et al., 2012).

Recent comparative studies of three *Brassicaceae* species, namely two halophytes, *Cakile maritime* and *Thellungiella salsuginea*, and a glycophyte, *Arabidopsis thaliana*, demonstrated that both osmotic potential changes and enhanced transient ROS production occurred in the halophyte roots within hours of exposure to NaCl, and subsequently enhanced the activation of antioxidant defenses as well as expression of transcription factors in the leaves and roots under long-term salinity stress. In contrast, the ROS accumulation continued in the glycophyte, *Arabidopsis*, during the entire observation period (Ellouzi et al., 2014), indicating that osmotic shock triggered by salinity stress may activate the initial production of ROS, and this phase is the basis of all functional changes leading to a second phase to activate defense mechanisms (Choudhury et al., 2013). The early production of H_2_O_2_ by RbohD and RbohF is suggested to be required for salinity-induced antioxidant defense responses in *Arabidopsis* under short-term stress treatments (Ben Rejeb et al., 2015). Interestingly, the Nox activity decreased under salinity stress in a glycophyte (*B. juncea*), while it was unaffected in a halophyte (*S. portulacastrum*) (Srivastava et al., 2015). These findings suggest that early production of low levels of H_2_O_2_ acts as an acclimation signal to trigger a preconditioning response by inducing antioxidant enzyme activities in order to efficiently cope with the subsequent production of ROS and lipid peroxidation, thereby preventing or minimizing salinity stress-derived injuries in several halophytes. Regulatory mechanisms of Nox-mediated ROS production in the salinity stress response have not yet been examined genetically in halophytes. Future studies on Rbohs/Noxs in various plant species including halophytes may reveal a novel function of ROS production as signaling molecules during salinity adaptation.

Halophytes do not always need to possess high antioxidant activity because of their higher capacity to prevent ROS generation (Bose et al., 2014). Salt-tolerant species equipped with efficient mechanisms for Na^+^ exclusion from the cytosol may not require a high level of antioxidant activity. Such exclusion is most probably achieved by the orchestrated action of several complementary mechanisms including SOS1-mediated Na^+^ exclusion from the cell (Shi et al., 2000), vacuolar Na^+^ sequestration by tonoplast NHXs (Blumwald, 2000) and efficient control of tonoplast fast (FV) and slow (SV) channels to prevent Na^+^ back-leak into the cytosol (Bonales-Alatorre et al., 2013a,b).

Although a rapid [Ca^2+^]_cyt_ increase mediated by PM Ca^2+^-permeable MS channels and their interacting molecules is a hallmark response to osmotic stress (Monshausen and Gilroy, 2009; Kurusu et al., 2013), Ca^2+^-independent osmotic sensory mechanisms may also play a role. Effectors of ROS and signaling cascades downstream of Rbohs need to be elucidated to better understand their roles. The crosstalk of Rboh/Nox-mediated Ca^2+^-dependent ROS production with other signaling pathways involving ABA, MAPK, and NO as well as jasmonic acid may be important in transcriptional regulation.

In addition, mild salinity stress in the seedling stage induces osmotic priming in *Arabidopsis* (Sani et al., 2013). Since salinity stress has been shown to affect DNA methylation status of many promoters in soybean (Song et al., 2012), the promoter regions of *Rboh* in halophytes may have characteristic consensus sequences involved in chromatin modification, and may, thus, enhance plant salinity tolerance.

## Conflict of Interest Statement

The authors declare that the research was conducted in the absence of any commercial or financial relationships that could be construed as a potential conflict of interest.

## Abbreviations

CaM: calmodulin
CBL: calcineurin B-like protein
CDPK: calcium-dependent protein kinase
CIPK: CBL-interacting protein kinase
CML: calmodulin-like protein
[Ca^2+^]_cyt_: cytosolic concentration of free Ca^2+^
HEK: human embryonic kidney
MCA: *mid1*-complementing activity
MS: mechanosensitive
MSL: MscS-like protein
Nox: NADPH oxidase
NSCC: non-selective cation channel
OSCA1: reduced hyperosmolarity-induced [Ca^2+^]_i_ increase 1
PCD: programmed cell death
PM: plasma membrane
Rboh: respiratory burst oxidase homolog
RLCK: receptor-like cytoplasmic kinase
ROS: reactive oxygen species
SOS: salt overly sensitive
TPC1: two-pore channel 1.
